# Modulation of Phytohormone Signaling: A Primary Function of Flavonoids in Plant–Environment Interactions

**DOI:** 10.3389/fpls.2018.01042

**Published:** 2018-07-20

**Authors:** Cecilia Brunetti, Alessio Fini, Federico Sebastiani, Antonella Gori, Massimiliano Tattini

**Affiliations:** ^1^National Research Council of Italy, Department of Biology, Agriculture and Food Sciences, Trees and Timber Institute, Florence, Italy; ^2^Department of Agri-Food Production and Environmental Sciences, University of Florence, Florence, Italy; ^3^Department of Agricultural and Environmental Sciences—Production, Landscape, Agroenergy, University of Milan, Milan, Italy; ^4^National Research Council of Italy, Department of Biology, Agriculture and Food Sciences, Institute for Sustainable Plant Protection, Florence, Italy

**Keywords:** flavonols, abscisic acid (ABA), auxin, reactive oxygen species (ROS), mitogen-activated protein kinases (MAPKs), early land plants

## Abstract

The old observation that plants preferentially synthesize flavonoids with respect to the wide range of phenylpropanoid structures when exposed to high doses of UV-B radiation has supported the view that flavonoids are primarily involved in absorbing the shortest solar wavelengths in photoprotection. However, there is compelling evidence that the biosynthesis of flavonoids is similarly upregulated in response to high photosynthetically active radiation in the presence or in the absence of UV-radiation, as well as in response to excess metal ions and photosynthetic redox unbalance. This supports the hypothesis that flavonoids may play prominent roles as scavengers of reactive oxygen species (ROS) generated by light excess. These ‘antioxidant’ functions of flavonoids appears robust, as maintained between different life kingdoms, e.g., plants and animals. The ability of flavonoids to buffer stress-induced large alterations in ROS homeostasis and, hence, to modulate the ROS-signaling cascade, is at the base of well-known functions of flavonoids as developmental regulators in both plants and animals. There is both long and very recent evidence indeed that, in plants, flavonoids may strongly affect phytohormone signaling, e.g., auxin and abscisic acid signaling. This function is served by flavonoids in a very low (nM) concentration range and involves the ability of flavonoids to inhibit the activity of a wide range of protein kinases, including but not limited to mitogen-activated protein kinases, that operate downstream of ROS in the regulation of cell growth and differentiation. For example, flavonoids inhibit the transport of auxin acting on serine–threonine PINOID (PID) kinases that regulate the localization of auxin efflux facilitators PIN-formed (PIN) proteins. Flavonoids may also determine auxin gradients at cellular and tissue levels, and the consequential developmental processes, by reducing auxin catabolism. Recent observations lead to the hypothesis that regulation/modulation of auxin transport/signaling is likely an ancestral function of flavonoids. The antagonistic functions of flavonoids on ABA-induced stomatal closure also offer novel hypotheses on the functional role of flavonoids in plant–environment interactions, in early as well as in modern terrestrial plants. Here, we surmise that the regulation of phytohormone signaling might have represented a primary function served by flavonols for the conquest of land by plants and it is still of major significance for the successful acclimation of modern terrestrial plants to a severe excess of radiant energy.

## Introduction

Flavonoids, a vast class of phenylpropanoids comprising more than 8,000 structures with a wide range of decorations, have the ability to absorb the most energetic solar wavelengths reaching the leaf surface (Agati and Tattini, 2010). Flavonoids have long been considered as primarily synthesized to constitute an effective shield against the penetration of UV-B radiation to sensitive leaf tissues, and greatly involved in protecting plants challenged by the depletion of stratospheric ozone layer (for a review, see Bais et al., 2018). Though this is obvious, as the UV-B (and UV-A) screening properties of flavonoids are long known, recent evidence suggests flavonoids may serve other ‘eco-physiological’ functions in early as well as in modern terrestrial plants (Pollastri and Tattini, 2011; Agati et al., 2012, 2013).

First, it is noted that all phenylpropanoids, not only flavonoids, have the ability to absorb wavelengths over the UV-B region of the solar spectrum. For instance, hydroxycinnamic acid derivatives have greater molar extinction coefficients (𝜀) than flavonoids over the UV-B waveband. Nonetheless, the biosynthesis of flavonoids occur at the expense of hydroxycinnamate biosynthesis in UV-B-treated leaves (Agati and Tattini, 2010; Agati et al., 2012, 2013).

Second, flavonoids have maximum 𝜀_s_ over the 330–355 nm, UV-A region of the solar spectrum, so that the induction spectrum most effective for their biosynthesis (UV-B) does not overlap with their absorption spectrum maxima, thereby suggesting that flavonoids are not synthesized to primarily serve UV-B screening functions in UV-B-treated tissues (Landry et al., 1995; Cockell and Knowland, 1999). This conforms to the observation that the biosynthesis of flavonoids is activated to a similar degree by high solar irradiance, in the presence or in the absence of UV-radiation (Agati et al., 2009, 2011). Moreover, blue light has been recently reported as being more effective than UV-B light in promoting the biosynthesis of flavonoids (Siipola et al., 2015). Finally, flavonoids accumulate to a very similar extent in plants exposed to excess both NaCl and Cu^2+^ or upon exposure to high UV-B irradiance (Babu et al., 2003; Agati et al., 2011). This is in line with the notion that changes in the redox potential of the cell activate flavonoid (particularly flavonol) biosynthesis (Taylor and Grotewold, 2005; Akhtar et al., 2010), consistent with relatively old findings that R2R3MYB transcription factors that regulate the biosynthesis of flavonoids, are themselves redox controlled (reviewed in Dubos et al., 2010).

Third, the biosynthesis of flavonoids with ortho-dihydroxy B-ring substitution is strongly favored as compared to the biosynthesis of monohydroxy B-ring-substituted flavonoids in response to excess light, irrespective of the relative proportions of solar wavelengths reaching the leaf surface (for a review, see Agati and Tattini, 2010). Monohydroxy and dihydroxy flavonoids do not display different UV-screening abilities, but largely differ in their ability to scavenge reactive oxygen species (ROS), i.e., to behave as antioxidants (Agati et al., 2012, 2013). This is in line with old and recent views that flavonoids may play major functions as antioxidants in plants exposed to excess light and, hence, to photooxidative stress of largely different origin (Landry et al., 1995; Ryan et al., 1998; Lillo et al., 2008; Agati et al., 2012; Tattini et al., 2015). It is a part of the folklore in plant photobiology and plant ecology that UV-B-treated leaves display a steeply higher quercetin to kaempferol (or apigenin) ratio than untreated leaves, and this is for equipping leaves with a more effective antioxidant, not UV-screening potential (Agati and Tattini, 2010; Agati et al., 2012).

The antioxidant properties of flavonoids have long been invoked to explain their health promoting effects in animals (Halliwell et al., 2005; Croft, 2016, for critical reviews), though flavonoid aglycones may also behave as pro-oxidants in high concentrations (Kessler et al., 2010). Since flavonoids are usually glycosylated in plant cells, thereby increasing their solubility and facilitating their transport from the endoplasmic reticulum (ER, the site of their biosynthesis) to different cellular organelles (Agati et al., 2012), their pro-oxidant actions are likely of minor significance in plants. There is an increasing body of evidence indicating that flavonoids may exert complex functions in both animal and plant cell metabolism, going well-beyond the mere chemical quenching of ROS. Certain flavonoids, especially but not limited to quercetin derivatives, have the ability to modulate signaling cascades that regulate cell growth and differentiation. For instance, flavonoids may strongly affect the protein kinase [e.g., mitogen-activated protein kinases (MAPKs)] signaling cascades (Williams et al., 2004; Hou and Kumamoto, 2010), which are at the base of many disorders, including carcinogenesis, in humans. In other words, flavonoids may behave as signaling molecules (Peer and Murphy, 2006), and in a concentration range much lower than that required to both scavenge ROS and effectively absorb UV-radiation (Agati and Tattini, 2010; Pollastri and Tattini, 2011). Though the ability of flavonoids to regulate cell growth and differentiation in plants has been known for decades (Jacobs and Rubery, 1988; Stafford, 1991), the functional significance in the adaptive mechanisms of plants to unfavorable habitats has been explored in less detail (Potters et al., 2009; Di Ferdinando et al., 2014).

Here, we focus our discussion on old and recent evidence of the strong relationship between flavonols (the ancient class of flavonoids already present in mosses, Wolf et al., 2010), auxin (IAA, Peer et al., 2013; Gayomba et al., 2017) and abscisic acid (ABA; Watkins et al., 2014, 2017). We hypothesize that the regulation of phytohormone signaling by flavonoids might have represented a function of primary significance for the conquest of land by plants and it is still of major value for the successful acclimation of modern terrestrial plants to a severe excess of radiant energy.

## Regulation of Phytohormone Signaling: a Robust Function of Flavonoids in Terrestrial Plants

One of the molecular innovations that accompanied the water-to-land transition of plants was the replacement of mycosporine like aminoacid (MAA) with the flavonoid, particularly flavonol metabolism (Cockell and Knowland, 1999). The functional reason(s) for such drastic metabolic modification was unlikely used for equipping early land plants with a more effective UV-B shield as compared to algae: MAA are at least as efficient as flavonoids to absorb UV-B radiation (Cockell and Knowland, 1999; Agati et al., 2013). The replacement of ‘nitrogen-rich’ with carbon-based UV-screening pigments might have been functional when early land plants moved to nutrient poor environments (Pollastri and Tattini, 2011). There is consensus that flavonols, particularly derivatives of quercetin, may have enhanced the ability of early land plants to take-up water and nutrients, acting on soil chemistry (Cesco et al., 2012) and, particularly by promoting the symbiosis with nitrogen-fixing bacteria and the plant–mycorrhiza association (Wasson et al., 2006; Hassan and Mathesius, 2012). The plant–mycorrhiza association represented a central event at the origin of land flora (Bonfante and Genre, 2008; Wang et al., 2010; Field et al., 2015). Notably, flavonols play the peculiar role of inhibiting auxin transport during nodulation (Zhang et al., 2009; Ng et al., 2015), thus determining increases in local auxin levels that, in turn, promote nodule organogenesis (Hassan and Mathesius, 2012). This adds further support to the old hypothesis that the origin of land plants resulted from “a union of alga and fungus advanced by flavonoids” Jorgensen (1993).

**FIGURE 1:**
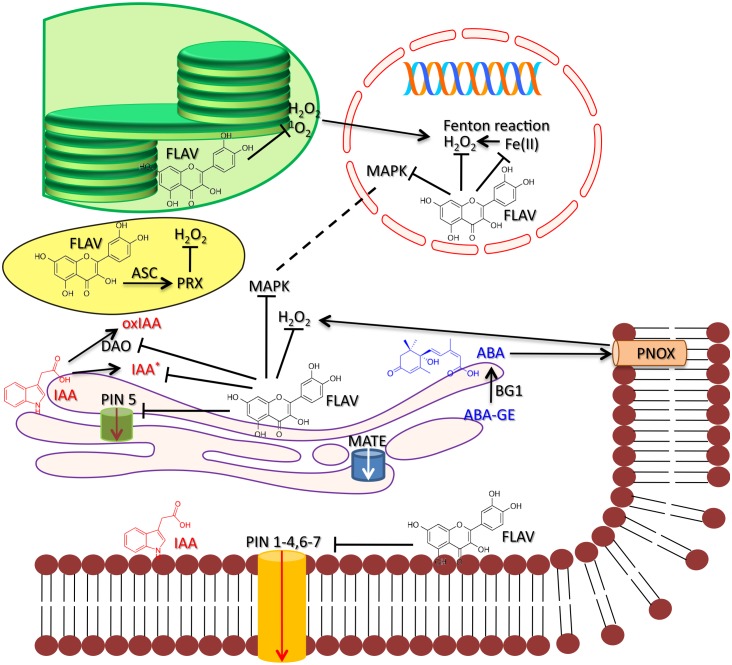
A proposed regulatory circuit involving auxin (IAA), abscisic acid (ABA), and flavonols (here represented by quercetin) under high light stress. High light activates the biosynthesis of IAA and ABA and, hence, the biosynthesis of flavonols. IAA is indeed synthesized at the cytoplasmic face of the ER, the very same site of flavonoid biosynthesis, and enhanced ABA biosynthesis under high light conditions mostly originates from de-glucosylation of ABA-GE, through the action of β-glucosidase1 (BG1) located at the ER (Lee et al., 2006; Tattini et al., 2017). The IAA- and ABA-induced flavonol biosynthesis might occur through the involvement of the bZIP transcription factor HY5 that, in turn, activates the expression of MYB12 (Lewis et al., 2011; Tossi et al., 2012). Flavonols distributed in different cellular compartments regulate the IAA and ABA-signaling. ER-located flavonoids may inhibit the activity of PIN5 (and perhaps of PIN6 and PIN8) auxin transport protein that escorts auxin into the ER lumen (Mravec et al., 2009). Flavonols are also transported to the ER lumen, by ABC-type and MATE proteins, and then to the plasma membrane (PM, Kitamura, 2006), where they inhibit the cell-to-cell auxin movement by acting on ‘long’ PINs (but also on PIN6, which has a dual, ER and PM localization, Simon et al., 2016). Flavonols may also alter the auxin catabolism by negatively affecting the activity of DIOXYGENASE for AUXIN OXIDATION (DAO), and hence the production of oxidized auxin (oxIAA, Zhang et al., 2016; Zhang and Peer, 2017), as well as by limiting the generation of IAA radicals (Mathesius, 2001). Chloroplast-located flavonols may complement the action of primary antioxidants (e.g., ascorbate peroxidase) the activity of which decreases under severe light excess (Mullineaux and Karpinski, 2002; Tattini et al., 2015). Flavonols may affect singlet oxygen (^1^O_2_) and H_2_O_2_-induced retrograde signaling, which, in turn may lead to programmed cell death (Agati et al., 2007, 2013; Fischer et al., 2007). This may occur through not only ROS scavenging, but also by strongly interacting with cytoplasmic- and nuclear-distributed MAPKs. Translocation of MAPKs from the cytoplasm to nucleus assists indeed cell re-programming under stressful conditions (Komis et al., 2018). Nuclear flavonols may indeed chelate transition metal ions (Hernández et al., 2009), such Fe (II), thereby preventing the massive generation of the highly reactive hydroxyl radical (OH^∙^) through the Fenton reaction [Fe(II) + H_2_O_2_ → Fe(III) + OH^∙^ + OH^-^]. Finally, vacuolar flavonols may scavenge H_2_O_2_ that freely escapes from the chloroplast at considerable rates under severe light excess (Mubarakshina et al., 2010), serving as substrates for vacuolar peroxidase and, then being recycled back to their original (reduced) forms by ascorbate (Sakihama et al., 2000). The tight control of cellular and inter-cellular auxin homeostasis by flavonols determines auxin gradients that regulate cell growth and differentiation, though we cannot exclude a direct involvement of nuclear-located flavonols on cell re-programming in response to severe light excess. Flavonols may scavenge H_2_O_2_, generated by the action of NADPH oxidase (Tossi et al., 2009), a key second *messenger in the ABA signaling network, as well as by possibly inhibiting the activity of MAPKs, which act downstream of H_2_O_2_ and are involved in the ABA-induced regulation of stomata movements (Jammes et al., 2009). This is consistent with flavonols being distributed in the cytoplasm, and mostly in the nucleus (Watkins et al., 2014), but not in the vacuole in stomata guard cells, at least in *Arabidopsis*.*

Flavonols are well-suited to modulate auxin transport and signaling, given their ability to affect the activities of a wide range of proteins (Jacobs and Rubery, 1988; Peer and Murphy, 2006; Santelia et al., 2008; Lewis et al., 2011; Peer et al., 2011), as well as to scavenge ROS (Tattini et al., 2004; Agati et al., 2007; Agati and Tattini, 2010; Peer et al., 2013). Ortho-dihydroxy B-ring-substituted flavonoids, such as quercetin, are particularly effective in affecting the activity serine–threonine PINOID (PID) proteins that control the localization of PINFORMED (PIN) auxin efflux facilitator proteins (PIN) proteins (**Figure 1**; Peer and Murphy, 2006, 2007; Michniewicz et al., 2007; Adamowski and Friml, 2015). Nonetheless, the significance of flavonoids as direct or indirect inhibitors of auxin transporters in the modulation of auxin gradients at cellular and inter-cellular levels and, hence, of auxin signaling has been questioned in some instances (e.g., Peer et al., 2011). Instead, flavonoids might determine gradients in auxin concentration at both cellular and tissue level by primarily modulating auxin catabolism, i.e., acting as direct and/or indirect scavengers of ROS (Jansen et al., 2001; Peer et al., 2011, 2013; Pollastri and Tattini, 2011; Zhang and Peer, 2017). However, how the ROS-scavenging capacities of flavonols may have an impact on auxin signaling is a matter that deserves further investigation (Gayomba et al., 2017). Antioxidant flavonoids may limit IAA-oxidation, by inhibiting the activity of DIOXYGENASE for AUXIN OXIDATION1 protein (DAO1, Zhang et al., 2016), a member of the 2-oxoglutarate and Fe(II)-dependent [2OG Fe(II)] oxygenase superfamily (**Figure 1**). Furthermore, flavonols may both reduce IAA radicals generated during IAA-oxidation and chelate Mn(II), which is a cofactor of IAA-oxidase (Mathesius, 2001). As noted above, this may be responsible for determining auxin gradients and the consequential developmental processes. There is evidence, however, that increased IAA levels may promote ROS formation and then stimulate IAA oxidation, thereby transiently repressing auxin signaling (Peer et al., 2013; Zhang and Peer, 2017). Flavonoids might act as general buffers of cellular ROS levels (though how and how much flavonoids may regulate the cytoplasmic ROS levels involved in IAA-oxidation and IAA-signaling is far from being fully elucidated (**Figure 1**; Gayomba et al., 2017), thus rendering plants in a “receptive state” capable to respond promptly to changes in environmental conditions (Peer et al., 2013). Similarly, stress-induced increase in ROS, e.g., H_2_O_2_, levels may activate specific MAPK, such as ANP1 kinase in *Arabidopsis* and NPK1 in tobacco which, in turn, repress auxin signaling while transducing oxidative stress signaling (Kovtun et al., 2000). In other words, the massive generation of H_2_O_2_ triggers ANP1-mediated MAPK cascade, and may help stressed plants to divert energy from auxin-related activities to stress protection (Kovtun et al., 2000).

We speculate that drastic changes in cellular redox homeostasis activate flavonoid (particularly flavonol) biosynthesis (Taylor and Grotewold, 2005; Akhtar et al., 2010; Dubos et al., 2010) and, in turn, antioxidant flavonoids might act as components of a regulatory circuit of auxin signaling pathway. It is worth noting that flavonoids accumulate greatly indeed in regions of high auxin concentration (Peer et al., 2004; Lewis et al., 2011; Grunewald et al., 2012). Further support to our hypothesis is the observation that antioxidant flavonoids have long been reported as having a nuclear location (and perhaps synthesized in the nucleus, for a review see Agati et al., 2012; Watkins et al., 2014) and, hence, optimally suited to strongly affect MAPK activities. This may be of great significance under stressful conditions, when MAPKs re-distribute from the cytoplasm to the nucleus, thus assisting cell re-programming (Komis et al., 2018; **Figure 1**).

There is relatively recent evidence that a subclade of PIN proteins, characterized by a shorter hydrophilic domain, such as PIN5, PIN6 and PIN8, as compared to ‘long’ plasma membrane (PM) PINs, are located at the ER (Mravec et al., 2009). Notably, the auxin metabolic pathway is also compartmentalized to the ER, as evidenced by the presence of several auxin metabolic enzymes and regulatory proteins in the ER (Bartel and Fink, 1995; Woodward and Bartel, 2005; Friml and Jones, 2010). These ER-located PIN proteins were already present in early land plants, such as *Physcomitrella patens* and *Selaginella moellendorffii*, thereby suggesting that mediating auxin homeostasis at the ER is the ancestral function of PIN proteins (Friml and Jones, 2010; Viaene et al., 2014). In particular, PIN5 escorts auxin from the cytoplasmic face of the ER (the site of auxin biosynthesis) to the ER lumen (**Figure 1**), therefore both increasing auxin compartmentation (Mravec et al., 2009; Kriechbaumer et al., 2012) and establishing auxin gradients at cellular level. Cell-to-cell auxin transport at the PM likely occurred at a later stage during land plant evolution and involved long PINs (e.g., PIN1 and PIN2; Friml and Jones, 2010; Peer et al., 2011; **Figure 1**). Notably, the cytoplasmic face of the ER is also the site of flavonoid biosynthesis (Burbulis and Winkel-Shirley, 1999), and both ATP binding cassette (ABC)-type and multidrug resistance and toxic ion extrusion (MATE) proteins assist the ‘transport’ of flavonoids to the ER lumen (reviewed in Kitamura, 2006; **Figure 1**). It is conceivable that the strong relationship between flavonoids (particularly flavonols) and auxin may constitute an ancestral feature of land plants, possibly contributing to enhance their ability to adapt, i.e., evolvability *sensu stricto* (Wagner, 2011; Lachowiec et al., 2016), to an ever-changing environment (Pollastri and Tattini, 2011).

We hypothesize that the regulation of auxin both movement and signaling, through the regulation of ROS levels and protein activities (**Figure 1**), i.e., signaling functions *sensu lato* (Peer and Murphy, 2006) was a function of primary significance served by flavonoids during the colonization of land by plants. These signaling functions are of key significance in the ecology of modern terrestrial plants as well, as capable of greatly affecting the organ as well as the whole-plant morphology. For instance, *Arabidopsis transparent testa (tt)* mutants, deficient in flavonoid biosynthesis, have elevated auxin transport and display phenotypes with largely impaired apical dominance (Brown et al., 2001). It has been speculated that UVR8-induced preferential activation of quercetin biosynthesis may well-contribute to the regulation of auxin signaling and the compact architecture (bushy phenotypes), of plants long-exposed to UV-B or full solar radiation (Tattini et al., 2000; Hectors et al., 2012; Hayes et al., 2014). Antioxidant flavonoids have the potential to strongly control the plant architecture (Jansen, 2002; Buer and Djordjevic, 2009), and likely play an important role in the stress-induced (particularly high light stress) morphogenic responses (SIMR), the flight strategy of sessile organisms (Tattini et al., 2000; Potters et al., 2007, 2009). It may be not a mere coincidence that environmental stimuli that result into a severe excess of radiant energy, and induce large alterations in the shape of individual organs and the whole-plant, almost exclusively promotes the biosynthesis of quercetin derivatives in all order of taxa, in the presence or in the absence of UV-radiation (Agati et al., 2012). This is also consistent with the observation that both the moss *P. patens* and the angiosperm *A. thaliana* respond very similarly to elevated doses of UV-B radiation, mostly enhancing the biosynthesis of quercetin derivatives (Wolf et al., 2010). There is very recent evidence indeed (Soriano et al., 2018) that (1) both the moss *P. patens* and the liverwort *M. polymorpha* have functional UVR8 proteins that are regulated similarly to the *Arabidopsis* UVR8, and (2) in both bryophytes, UVR8 proteins mediate *HY5* (*ELONGATED HYPOCOTYL5*) transcript expression and CHS (CHALCONE SYNTHASE) protein accumulation in response to UV-B. Notably, *HY5* regulates the expression of *MYB12* and *MYB111* genes, known as *PRODUCTION OF FLAVONOL GLYCOSIDES* (*PFG)*, in response to not only UV-B, but also to high white light (Stracke et al., 2010).

There is scarce evidence of PIN-flavonoid modulation of plant shape in bryophytes. However, recent findings show that the ‘ancestral’ PIN6 protein (Friml and Jones, 2010) in *Arabidopsis* (Simon et al., 2016) and PINA in *P. patens* have dual ER and PM localization. The finding that PINs proteins in *P. patens* are highly responsive to naringenin and strongly controls the shoot architecture (Bennet et al., 2014) is of significance, and opens new perspectives on similar regulation of plant shape by PIN/flavonoids in bryophytes and angiosperms.

There is recent compelling evidence that flavonols, especially derivatives of quercetin may greatly affect the ABA-signaling pathway, by antagonizing the ABA-induced stomatal closure in both *Arabidopsis* and tomato (Watkins et al., 2014, 2017). *Arabidopsis* guard cells rich in quercetin have reduced levels of H_2_O_2_ and, consequently, greater stomatal aperture than stomata deprived of quercetin (Watkins et al., 2014). Similarly, the *anthocyanin reduced* (*are*) tomato mutant, which has low flavonol levels, displays higher ROS content and lower stomatal aperture as compared to the *anthocyanin without* (*aw*) mutant, which is rich in quercetin (Watkins et al., 2017). H_2_O_2_ is a key second messenger in the ABA-signaling pathway, and it is necessary to promote stomatal closure (reviewed in Wang and Song, 2008). It is conceivable that the antagonistic effect of quercetin on ABA-signaling might result not only from its ability to quench H_2_O_2_, but also through the inhibition of MAPKs activities, which act downstream of H_2_O_2_ in the regulation of guard cell movements (Jammes et al., 2009; Danquah et al., 2013; **Figure 1**). Our hypothesis is strongly supported by the observation that flavonols in *Arabidopsis* guard cells have cytoplasmic and especially nuclear distribution (Watkins et al., 2014).

Notably, ABA has also been reported to promote the biosynthesis of flavonols (Berli et al., 2010, 2011), other than the biosynthesis of anthocyanins (Shen et al., 2014; Kadomura-Ishikawa et al., 2015). This is not surprising, as relatively recent experiments have shown a strong integration between ABA and light signaling (Bechtold et al., 2008; Wang et al., 2017), which may also help to explain the steeply enhanced flavonol biosynthesis in leaves exposed to high light irradiance, even in the absence of UV-radiation. There is intriguing evidence that increases in the levels of foliar ABA upon high light irradiance likely results from enhanced de-glucosylation of the ‘inactive’ ABA-glucoside (ABA-GE), and not from *de novo* synthesis through the plastidial MEP-pathway (Lee et al., 2006; Tattini et al., 2017). The notion that β-glucosidase1 (BG1), which promotes the release of free-ABA from ABA-GE, is located at the ER (Lee et al., 2006), the site of flavonoid biosynthesis, is of the greatest interest. We speculate that the quick production of free ABA from ABA-GE (Leng et al., 2014) might sustain steep increases in local ABA concentrations that are required for initiating early signaling responses to excess light stress, and these may well-include the biosynthesis of flavonols (**Figure 1**). The flavonol regulation of the ABA-signaling pathway may offer an additional chemical control to fine tune stomata movement. Nonetheless, how and how much the flavonols–ABA relationship may affect the acclimation/adaptive responses of plants to environmental pressures associated to habitats with contrasting soil water availability and solar irradiance is an interesting matter that needs further investigation. It is, however, plausible that the flavonol-regulation of stomata movements may have allowed early land plants to radiate toward habitats of increasingly excess of solar irradiance and contrasting soil water availability, thereby contributing to their adaptation on land.

## Concluding Remarks

Here, we have hypothesized that the mutual regulation of phytohormone signaling and flavonol, particularly quercetin biosynthesis may have been of great value for the adaptation of plants on the land. It may be not a mere coincidence that mosses as well as modern terrestrial plants preferentially synthesize derivatives of quercetin in response to a severe light excess, despite the evolution of flavonoid metabolism has produced 1000s of structures. This is likely for equipping plants with a versatile metabolite capable of serving multiple functions (Pollastri and Tattini, 2011). Here we have shown that quercetin has a superior ability to modulate ROS- and phytohormone signaling, but not a greater capacity to screen-off the most energetic (UV-B) solar wavelengths as compared to other flavonoids. Our reasoning supports the view that modulation of ROS and phytohormone signaling is likely an ancestral, robust function of quercetin (as well as of other ‘antioxidant’ flavonoids), since it has a persisted against environmental, stochastic and genetic perturbations, over an extended time-scale level (Felix and Wagner, 2008; Lachowiec et al., 2016).

The observation that a wide range of abiotic stressors activates the biosynthesis of flavonoids, particularly of the di-hydroxy B-ring-substituted structures when plants suffer from severe excess of radiant energy, suggests that change in cellular ROS homeostasis is one of the main drivers for flavonoid biosynthesis (Taylor and Grotewold, 2005). In turn, flavonoids with effective antioxidant potential (i.e., they are ROS scavengers at very low concentrations, Asensi-Fabado and Munné-Bosch, 2010) may act as buffers of ROS levels, and enhance stress resistance. The ability of flavonoids to modulate ROS as well as phytohormone signaling (**Figure 1**) may help to explain their involvement in stress-induced morphogenic responses, a general response of plants to ‘fly away’ from adverse environmental conditions. For instance, ‘effective antioxidant’ flavonoids, distributed in the nucleus, the chloroplast, and the vacuole of mesophyll cells, constitutes more than 90% of the total flavonoid pool in plants inhabiting harsh, sunny habitats, and display bushy phenotypes, with small and thick leaves and short internodes. In contrast, monohydroxy-substituted, ‘poor antioxidant’ flavones and flavonols, mostly located in the adaxial epidermal cells, predominate in shade-adapted plants, whose phenotypes are associated to large, thin leaves and long internodes. These are, however, merely correlative observations, and further experimentation is needed to explore the significance of flavonol-phytohormone interactions in the mechanisms regulating the morphology and anatomy of individual organs and the whole plant under natural conditions.

## Author Contributions

All authors listed have made a substantial, direct and intellectual contribution to the work, and approved it for publication.

## Conflict of Interest Statement

The authors declare that the research was conducted in the absence of any commercial or financial relationships that could be construed as a potential conflict of interest.
